# Practice-Level Variation in Telemedicine Use in a Pediatric Primary Care Network During the COVID-19 Pandemic: Retrospective Analysis and Survey Study

**DOI:** 10.2196/24345

**Published:** 2020-12-18

**Authors:** Kelsey Schweiberger, Alejandro Hoberman, Jennifer Iagnemma, Pamela Schoemer, Joseph Squire, Jill Taormina, David Wolfson, Kristin N Ray

**Affiliations:** 1 Department of Pediatrics University of Pittsburgh School of Medicine UPMC Children's Hospital of Pittsburgh Pittsburgh, PA United States; 2 UPMC Children's Community Pediatrics Pittsburgh, PA United States

**Keywords:** telehealth, telemedicine, ambulatory pediatrics, health services research, ambulatory, pediatrics, health services, COVID-19

## Abstract

**Background:**

Telehealth, the delivery of health care through telecommunication technology, has potential to address multiple health system concerns. Despite this potential, only 15% of pediatric primary care clinicians reported using telemedicine as of 2016, with the majority identifying inadequate payment for these services as the largest barrier to their adoption. The COVID-19 pandemic led to rapid changes in payment and regulations surrounding telehealth, enabling its integration into primary care pediatrics.

**Objective:**

Due to limited use of telemedicine in primary care pediatrics prior to the COVID-19 pandemic, much is unknown about the role of telemedicine in pediatric primary care. To address this gap in knowledge, we examined the association between practice-level telemedicine use within a large pediatric primary care network and practice characteristics, telemedicine visit diagnoses, in-person visit volumes, child-level variations in telemedicine use, and clinician attitudes toward telemedicine.

**Methods:**

We analyzed electronic health record data from 45 primary care practices and administered a clinician survey to practice clinicians. Practices were stratified into tertiles based on rates of telemedicine use (low, intermediate, high) per 1000 patients per week during a two-week period (April 19 to May 2, 2020). By practice tertile, we compared (1) practice characteristics, (2) telemedicine visit diagnoses, (3) rates of in-person visits to the office, urgent care, and the emergency department, (4) child-level variation in telemedicine use, and (5) clinician attitudes toward telemedicine across these practices.

**Results:**

Across pediatric primary care practices, telemedicine visit rates ranged from 5 to 23 telemedicine visits per 1000 patients per week. Across all tertiles, the most frequent telemedicine visit diagnoses were mental health (28%-36% of visits) and dermatologic (15%-28%). Compared to low telemedicine use practices, high telemedicine use practices had fewer in-person office visits (10 vs 16 visits per 1000 patients per week, *P*=.005) but more total encounters overall (in-office and telemedicine: 28 vs 22 visits per 1000 patients per week, *P*=.006). Telemedicine use varied with child age, race and ethnicity, and recent preventive care; however, no significant interactions existed between these characteristics and practice-level telemedicine use. Finally, clinician attitudes regarding the usability and impact of telemedicine did not vary significantly across tertiles.

**Conclusions:**

Across a network of pediatric practices, we identified significant practice-level variation in telemedicine use, with increased use associated with more varied telemedicine diagnoses, fewer in-person office visits, and increased overall primary care encounter volume. Thus, in the context of the pandemic, when underutilization of primary care was prevalent, higher practice-level telemedicine use supported pediatric primary care encounter volume closer to usual rates. Child-level telemedicine use differed by child age, race and ethnicity, and recent preventive care, building upon prior concerns about differences in access to telemedicine. However, increased practice-level use of telemedicine services was not associated with reduced or increased differences in use, suggesting that further work is needed to promote equitable access to primary care telemedicine.

## Introduction

Telehealth, the delivery of health care through telecommunication technology, has potential to address multiple health system concerns; it can alleviate physician workforce shortages, improve access to care, mitigate disparities in health care, control costs, and enhance communication between clinicians [1-3]. Despite this potential, the uptake of telehealth among pediatric clinicians has largely remained outside of primary care pediatrics, with growth instead observed in mental health, subspecialty care, and direct-to-consumer telemedicine provided by clinicians outside of the medical home [4,5]. The American Academy of Pediatrics (AAP) cautions against pediatric telemedicine provided outside of the primary care office due to concerns about fragmentation of care, suboptimal care quality, and lack of integrated follow-up; however, the AAP supports integration of telehealth into primary care pediatrics within the patient-centered medical home [1,6].

Despite the AAP endorsement of telehealth within primary care, only 15% of pediatric primary care clinicians reported using telemedicine as of 2016, with the majority identifying inadequate payment for these services as the largest barrier [7]. As of February 2020, all state Medicaid programs had payment provisions for live video telehealth services; however, only 19 states paid for telehealth services when the patient was located in their home, and only 5 states mandated payment parity with in-person visits [8]. This situation was reflected in similar stipulations by commercial payers. With limited payment options for telehealth services, especially for patients located at home, the adoption of telemedicine was not financially viable for most pediatric primary care offices outside of integrated care delivery systems before March 2020 [7,9].

In March 2020, the COVID-19 pandemic precipitated a rapid need for increased telehealth services to safely deliver care while limiting the risk of exposure to contagion that is inherent in an in-person setting [10]. The nationwide need for telehealth services led to rapid changes in payment and regulations surrounding telehealth delivery. Specifically, updated policies allowed patients to be located in their homes during a telemedicine visit and allowed use of widely available technology platforms to deliver telehealth by waiving penalties for Health Insurance Portability and Accountability Act (HIPAA) violations [11,12]. These policy changes, and the shifting perceptions of risk versus benefit of in-person and virtual care, enabled the sudden adoption of telemedicine within primary care practices across the country [13-16].

Thus, we are witnessing an acute surge in telemedicine use within pediatric primary care; however, much is unknown about the potential uses and impact of telehealth in pediatric primary care, given the prior rarity of this model of care. In this paper, we describe the experience of a large pediatric primary care network within the first two months of telemedicine use during the COVID-19 pandemic. Specifically, we aimed to identify high versus low telemedicine-using primary care practices and compare (1) practice characteristics, (2) telemedicine visit diagnoses, (3) rates of in-person visits to the office, urgent care, and the emergency department (ED), (4) child-level variation in telemedicine use, and (5) clinician attitudes toward telemedicine across these practices.

## Methods

### Context and Study Population

We performed a retrospective analysis of electronic health record (EHR) data from 45 practices within a large pediatric primary care network. These practices are certified as patient-centered medical homes by The Joint Commission; together, they provide care for approximately 315,000 children throughout Western Pennsylvania across 13 counties. All the practices shared one EHR, which offered embedded video visits through a patient portal. Some of these practices had briefly trialed a model of acute care telemedicine in 2015; however, none of the practices were offering telemedicine services at the start of the pandemic.

### Telemedicine Implementation

Local payers began to offer payment for telemedicine when the patient is at home on March 17, 2020 [17]. On March 23, 2020, the first county-specific stay-at-home order was issued in Pennsylvania [17,18]. Several practice leads trialed multiple telemedicine platforms and workflows from March 18-20, 2020, with implementation strategies shared with all practices via videoconference on March 23, 2020. The network quality and safety leaders led collaborative learning videoconferences two to three times per week for the next two months with all physicians, advanced practice providers (APPs), and practice managers, sharing telemedicine best practices and discussing other COVID-19–related topics. Initial relaxation of the stay-at-home order for the largest metropolitan county occurred on May 15, 2020, with transition of the state to its “yellow phase.”

### Data Source

We obtained encounter data for all telemedicine and in-person visits between March 18 and May 2, 2020, from the EHR, and we identified patient panels for each practice, defined as all patients with one or more encounters at the practice in the prior two years. For each practice’s patient panel, patient demographics, date of last preventive visit, and counts of telemedicine, office, ED, and urgent care visits were obtained from the EHR. To complement this EHR-based evaluation, we also surveyed the primary care clinicians across the practice network regarding the usability, usefulness, and perception of patient and clinician experience of telemedicine.

### EHR Data and Variables

For each practice in the network, the number of practice clinicians (doctors and APPs) and practice site locations were obtained from network records. Practice site counties were classified as rural or urban using the 2013 rural-urban continuum codes [19].

Telemedicine visits were identified using EHR encounter type codes. All completed telemedicine visits from March 18 to May 2, 2020, were included. For each telemedicine visit, we extracted the practice site, age of the child on the date of visit, and primary visit diagnosis.

Telemedicine visit primary diagnoses were categorized based on International Classification of Diseases, Tenth Revision, Clinical Modification (ICD-10-CM) diagnosis codes into 22 broad ICD-10-CM diagnostic categories corresponding to organ systems [20]. For nonspecific categories (eg, “symptoms, signs, and abnormal clinical and laboratory findings, not elsewhere classified” (R00-R99)), we reviewed the subcategories and recategorized them into the relevant organ system. For example, the subcategory of “symptoms and signs involving the skin and subcutaneous tissue” (R20-R23) was grouped with “diseases of the skin and subcutaneous tissues” (L00-L99) into “skin and subcutaneous tissue diagnoses.”

To compare the volumes of telemedicine visits with other modes of care delivery, we extracted each practice’s volumes from the EHR, including in-person office, urgent care, and ED visits, as well as telephone encounters (excluding those with a telemedicine or in-person visit on the same day) during a 2-week window during the pandemic (April 19 to May 2, 2020).

Finally, for each child identified as part of a practice’s panel, we extracted age, race and ethnicity, health insurance (Medicaid vs commercial), and whether the child had a preventive visit within the prior 12 months. The child’s race and ethnicity were originally recorded in the EHR based on parent response during the child’s first visit with the practice. Across all practices, 82% of patients were identified as White non-Hispanic; therefore, analyses by patient race and ethnicity were limited to comparing children identified as White non-Hispanic to children identified as any other racial or ethnic identity, of which the majority identified as “other” (9%), non-Hispanic Black (8%), or Hispanic (1%).

### Clinician Survey

To complement this primarily EHR-based analysis, clinicians in the primary care network were invited to participate in a web-based survey. The survey items examined the usability and usefulness of telemedicine through items modified from the Technology Usability Questionnaire, which is a validated survey incorporating questions from the Technology Acceptance Model, Telemedicine Satisfaction Questionnaire, and Post-Study System Usability Questionnaire and encompasses five subscales: usefulness, ease of use, effectiveness, reliability, and satisfaction, with all items using Likert scales [21]. We added questions pertaining to the physician experience of telemedicine, including perceived impact on quality of care (informed by the Institute of Medicine’s six domains of quality), impact on job satisfaction (informed by self-determination theory), and perceived usefulness of telemedicine for different visit reasons [22,23]. The survey included 37 questions, and it is available in Multimedia Appendix 1. Participants had the option to identify their practice or leave this item blank.

Clinicians were invited to participate in the anonymous web-based survey from April 28 to May 14, 2020, through a series of 4 emails. The timeframe was chosen to capture summative attitudes and experiences of clinicians coinciding with the end of the EHR-based analysis.

### Identification of Low, Intermediate, and High Telemedicine Use Practices

For each practice, to account for variation in practice size, we determined the total number of telemedicine visits completed per week and divided the number of visits by the number of active patients in that practice to provide a standardized rate of telemedicine visits per 1000 patients. To categorize high versus low telemedicine use practices, the rate of telemedicine visits per 1000 patients per week was averaged for the 2-week period (April 19 to May 2, 2020) occurring after the first month of telemedicine implementation. This time frame was chosen to categorize practices at a time where telemedicine visit volume had stabilized so that the analysis could focus on practices with high versus low use (as opposed to early vs late adopters). The 45 practices were categorized into tertiles based on their telemedicine visit rates, which we labeled as low, intermediate, or high telemedicine use practices.

### Statistical Analysis

The analyses compared practice-level characteristics, telemedicine visit diagnoses, in-person visit volumes, variation in volume by patient characteristics, and clinician attitudes across telemedicine use tertiles. Across practice-level telemedicine use tertiles, we compared practice-level characteristics and in-person visits per 1000 patients per week using Kruskal-Wallis tests. We compared the percentage of telemedicine visits within each individual diagnosis category across tertiles using logistic regression.

For patients in each practice’s panel, we determined the percentage of children who had one or more telemedicine visits during the 2-week period of the focused analysis (April 19 to May 2, 2020) by specific child characteristics (age, race and ethnicity, insurance type, and receipt of a preventive visit in the prior year). First, to assess whether there was a significant difference in telemedicine use by child characteristics across all practices (regardless of practice-level telemedicine use), we used a child-level logistic regression model clustered by practice but with no tertile variable. To determine whether increased practice-level use of telemedicine altered the differences in telemedicine use by child characteristics, we assessed the significance of an interaction term between each child characteristic category and practice tertile within a series of child-level logistic regression models clustered by practice across all tertiles. We tested the significance of the interaction terms using Wald tests, and we present the results as adjusted percentages determined through these models.

Clinician survey responses were compared across tertiles using linear regression clustered by practice, excluding respondents who did not identify their practice. We also compared responses for respondents who did not identify their practice versus those who did, again using linear regression, and found no significant differences.

All analyses were conducted in Stata version 16.1 (StataCorp) with significance assessed using an alpha level of .05.

### Approval and Ethical Considerations

This analysis was part of a quality improvement project aimed at improving pediatric primary care telemedicine delivery and was approved by the University of Pittsburgh Medical Center’s Quality Review Committee. Projects approved by this committee do not meet the definition of human subjects research and therefore do not require formal approval by an institutional review board.

## Results

**Figure 1:**
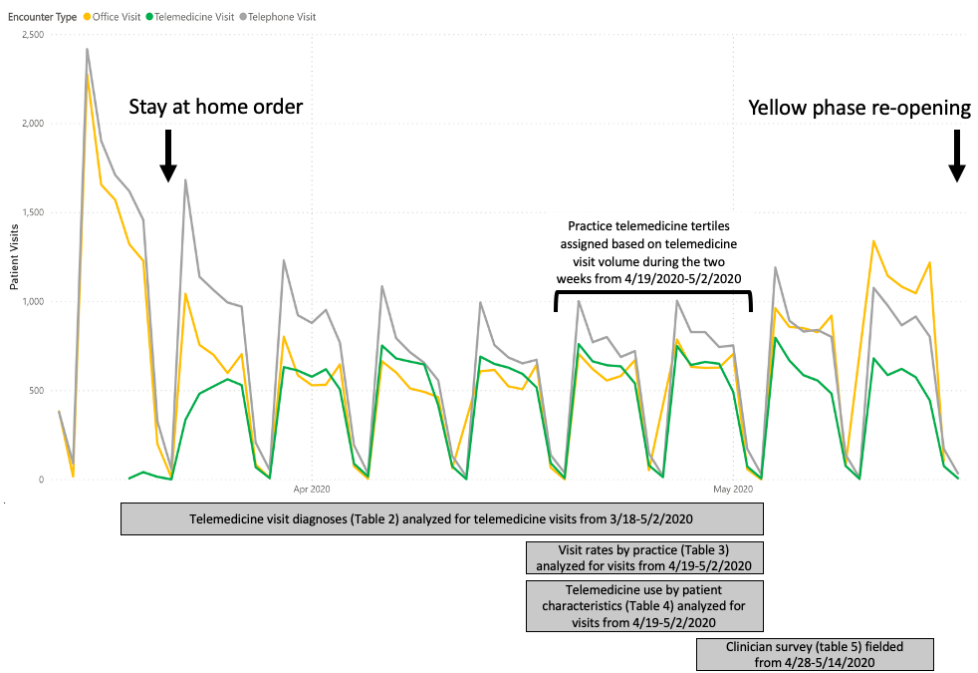
Encounters by telemedicine, in-person office, and care via telephone across 45 practices within a pediatric primary care network, March through May 2020. Telemedicine use tertiles were defined based on visit volume from April 19 to May 2, 2020 (indicated by brackets), as this period represented steady-state telemedicine use. Yellow phase indicates the first relaxation of the stay-at-home order, indicating the first phase of reopening in Pennsylvania.

Starting on March 23, 2020, the network underwent a rapid reduction of in-person office visit volumes and a simultaneous increase in telemedicine visit volumes (Figure 1). Telemedicine visit volume reached a steady state three to four weeks after implementation.

### Practice-Level Characteristics

High telemedicine use practices had more physicians in the practice (median 4) than low telemedicine use practices (median 3, *P*=.04); however, all other practice-level characteristics were similar (Table 1).

**Table 1:** Practice characteristics by low, intermediate, or high telemedicine use (N=45).

Variable			Telemedicine use			*P* value	
			Low	Intermediate	High		
Practices, n (%)			15 (33)	15 (33)	15 (33)	N/A^a^	
Telemedicine visits per 1000 patients per week, range			5-9.6	9.7-14	15-23	N/A	
**Practice characteristics**							
	Active patients in practice, range		1200-8300	2600-7800	2000-10400	.08	
	**Practice clinicians**						
		Physicians, median (IQR)	2 (1-6)	5 (2-10)	6 (4-10)	.04	
		APPs^b^, median (IQR)	2 (1-3)	2 (2-6)	2 (1-5)	.70	
	Percent of patient population insured by Medicaid, median (IQR)		61 (50-77)	62 (53-73)	79 (62-82)	.07	
	**Practice site (rural/urban)^c^**						
		Rural county, n (%)	4 (27)	4 (27)	3 (20)	.90	

^a^N/A: not applicable.

^b^APPs: advanced practice providers.

^c^Based on US Department of Agriculture rural-urban continuum codes.

### Telemedicine Visit Diagnoses

Across all tertiles, telemedicine visits were most common for mental health and skin/soft tissue–related diagnoses (Table 2). However, the percentage of visits in each of these diagnostic categories varied with practice-level telemedicine use. Visits with skin-related diagnoses, for example, comprised 17.5% of telemedicine visits (467/2661) at low telemedicine use practices, and 15.0% of visits (1435/9587) at high telemedicine use practices (*P*=.006). Although visits for skin-related diagnoses represented a smaller percentage of telemedicine visits at high telemedicine use practices, the number of skin-related visits per 1000 patients per week was higher at high telemedicine use practices compared to low telemedicine use practices (median 14 vs 7 telemedicine skin related visits per 1000 patients per week at high and low telemedicine use practices, respectively; *P*<.001). High telemedicine use practices had larger percentages of telemedicine visits devoted to respiratory (*P*<.001), ear/mastoid (*P*<.001), and genitourinary (*P*=.02) diagnoses than low telemedicine use practices (Table 2).

**Table 2:** Telemedicine visit diagnoses in practices (N=45) with low, intermediate, and high telemedicine use from March 18 to May 2, 2020.

Variable		Telemedicine use			*P* value^a^
		Low	Intermediate	High	
Telemedicine visits per 1000 patients per week, range		5-9.6	9.7-14	15-23	N/A^b^
Number of practices, n (%)		15 (33.3)	15 (33.3)	15 (33.3)	N/A
**Visit diagnosis category^c^ (total visits), n (%)**					
	Mental, behavioral, and neurodevelopmental diagnoses	969 (36.4)	1704 (31.9)	2724 (28.4)	<.001
	Skin and subcutaneous tissue diagnoses	467 (17.5)	801 (15.0)	1435 (15.0)	.006
	Respiratory system diagnoses	297 (11.2)	684 (12.8)	1395 (14.6)	<.001
	Digestive system diagnoses	182 (6.8)	396 (7.4)	697 (7.3)	.60
	Infectious and parasitic diagnoses	176 (6.6)	390 (7.3)	746 (7.8)	.04
	Injury/poisoning	92 (4)	184 (3.4)	437 (4.6)	.001
	General symptoms and signs	77 (3.5)	241 (4.5)	416 (4.3)	.01
	Eye diagnoses	77 (3.5)	147 (2.8)	255 (2.7)	.50
	Ear and mastoid process diagnoses	45 (1.7)	230 (4.3)	395 (4.1)	<.001
	Genitourinary system diagnoses	45 (1.7)	123 (2.3)	240 (2.5)	.02
	Other^d^	234 (8.8)	435 (8.2)	847 (8.8)	.60

^a^*P* values indicate significance of proportion of telemedicine visits within each individual diagnosis category compared across practice-level telemedicine use tertiles using logistic regression.

^b^N/A: not applicable.

^c^Categories based on codes in the International Classification of Diseases, Tenth Revision, Clinical Modification.

^d^Diagnostic categories with less than 2% of visits are represented in this “other” category; these included neoplasms and hematologic diagnoses, endocrine, nutritional and metabolic diseases, nervous system diagnoses, circulatory system diagnoses, musculoskeletal diagnoses , peripartum and perinatal diagnoses, congenital anomalies, and symptoms and signs not otherwise classified, as well as codes for special purposes, injuries, and contact with health services.

### In-Person Visit Volume

Compared to low telemedicine use practices, practices with high telemedicine use had fewer in-person office visits (median 10 vs 16 in-person office visits per 1000 patients per week at high vs low telemedicine use practices, respectively; *P*=.005; Table 3). Practices with high telemedicine use also had slightly more ED visits (median 2 vs 1 ED visits per 1000 patients per week at high and low telemedicine use practices, respectively; *P*=.02) but similar urgent care visits. When accounting for both in-person and telemedicine office visits, high telemedicine use practices had more total primary care encounters per 1000 patients per week (median 28 vs 22 encounters, *P*=.006). Of note, among high telemedicine use practices, total primary care encounters (in-person and telemedicine) represented a median of 53% of the weekly volume in 2019. In contrast, among low telemedicine use practices, total primary care encounters represented a median of 46% of the 2019 weekly volume. Telephone encounters occurring separate from a visit were similar across tertiles.

**Table 3:** In-person visits by patients in practices with low, intermediate, or high telemedicine use (April 19 to May 2, 2020).

		Telemedicine use			*P* value^a^
		Low	Intermediate	High	
Practices, n (%)		15 (33)	15 (33)	15 (33)	N/A^b^
**Primary care patient visits**					
	Telemedicine visits per 1000 patients per week, range	5-9.6	9.7-14	15-23	N/A
	In-person office visits per 1000 patients per week, median (IQR)	16 (12-18)	11 (7-14)	10 (8-12)	.005
	All primary care encounters per 1000 patients per week, median (IQR)	22 (19-26)	23 (19-26)	28 (25-30)	.006
**In-person patient visits outside of primary care, median (IQR)**					
	Urgent care visits per 1000 patients per week	0.4 (0-1)	1 (0.5-2)	1 (0.8-2)	.10
	Emergency department visits per 1000 patients per week	1 (0.8-1)	2 (1-2)	2 (1-2)	.02
	Total encounters per 1000 patients per week outside of primary care	2 (1-3)	3 (2-4)	3 (2-4)	.008
Total encounters per 1000 patients per week		25 (20-28)	25 (24-29)	30 (28-33)	.003
**Patient telephone calls to practice not associated with a visit, median (IQR)**					
	Telephone management without a visit per 1000 patients per week	17 (8-22)	14 (10-16)	15 (7-20)	.80

^a^*P* values indicate practice-level in-person visits per 1000 patients per week compared across practice-level telemedicine use tertiles using Kruskal-Wallis tests.

^b^N/A: not applicable.

### Patient Characteristics Associated With Telemedicine Use

In logistic regression models without practice-level tertile designation, use of telemedicine varied significantly by child race and ethnicity (*P*<.001), child age (*P*<.001), and receipt of preventive care in the prior 12 months (*P*<.001) but did not vary by child insurance category (*P*=.40). However, the interaction terms between the practice tertile and each of these characteristics were not significant (Table 4). This indicates that although differences exist in the full sample for telemedicine use by child race and ethnicity, child age, and recency of a preventive visit, increasing practice-level use of telemedicine did not change the relationship between these patient characteristics and the likelihood of a telemedicine visit.

**Table 4:** Variation in telemedicine visits by child characteristic across practices with low, intermediate, and high telemedicine use. Adjusted percentages of children in patient panels who had one or more telemedicine visits during the study period using logistic regression clustered by practice. Italicized variables are significant in the entire population. *P* values indicate the significance of the interaction term between the specified characteristic and practice tertile.

Model with no interaction term				Model with tertile and telemedicine use interaction term					
			All practices		Low telemedicine use practices	Intermediate telemedicine use practices	High telemedicine use practices		*P* for interaction term
Practices, n (%)			45 (100)		15 (33.3)	15 (33.3)	15 (33.3)		N/A^a^
Telemedicine visits per 1000 patients per week, range			5-23		5-9.6	9.7-14	15-23		N/A
Children, n (%)			244,473 (100)		66,295 (27.1)	84,093 (34.4)	94,085 (38.5)		N/A
**Adjusted percentage of children with one or more telemedicine visit, % (95% CI)**									
	***By child age (years)^b^***							.25	
		0-2	2.3 (2-2.7)		1.6 (0.8-2.3)	2.2 (1.9-2.6)	3.1 (2.6-3.5)		
		3-5	1.3 (1.1-1.5)		1 (0.7-1.4)	1 (0.9-1.2)	1.7 (1.4-2)		
		6-11	1.6 (1.5-1.8)		1.2 (0.9-1.4)	1.6 (1.5-1.8)	2 (1.8-2.2)		
		12-17	1.6 (1.4-1.7)		1.1 (0.9-1.3)	1.6 (1.3-1.9)	1.9 (1.7-2)		
	***By race/ethnicity***							.10	
		Non-Hispanic Black, Hispanic, and other race and ethnicity categories	1.4 (1.2-1.6)		0.7 (0.3-1.1)	1.3 (1.2-1.5)	1.8 (1.6-2.1)		
		Non-Hispanic White	1.7 (1.6-1.9)		1.2(1-1.5)	1.7 (1.5-1.8)	2.1 (2-2.3)		
	***By receipt of preventive care within the last year***							.26	
		Received preventive care in the last year	1.8 (1.7-2)		1.3 (1-1.6)	1.7 (1.6-1.9)	2.3 (2.1-2.5)		
		Did not receive preventive care in the last year	1.1 (0.9-1.2)		0.7 (0.6-0.8)	1.1 (1-1.2)	1.3 (1.1-1.5)		
**By insurance type^c^**								.2	
	Medicaid-insured children		1.7 (1.5-1.9)		1.2 (0.9-1.6)	1.6 (1.4-1.8)	2.3 (2-2.6)		
	Commercially insured children		1.6 (1.5-1.8)		1.1 (0.8-1.4)	1.6 (1.5-1.7)	2 (1.8-2.2)		

^a^N/A: not applicable.

^b^Patients over 18 years of age were excluded (n=20,424).

^c^Children whose insurance type was identified as self-pay were excluded (n=9258).

### Attitudes of Clinicians Toward Telemedicine

The survey was completed by 121 clinicians, including 88 who identified their practice and 33 who did not (34% response rate). Clinician attitudes regarding the usability, usefulness for child health, usefulness for clinician experience, or impact of telemedicine did not vary significantly across tertiles (Table 5).

**Table 5:** Clinician perceptions of usability and usefulness of telemedicine.

Variable			Clinicians at low telemedicine use practices		Clinicians at intermediate telemedicine use practices		Clinicians at high telemedicine use practices		*P* value^a^
Telemedicine visits per 1000 patients per week, range			5-9.6		9.7-14		15-23		N/A^b^
Clinicians, n (%)			21 (24)		35 (40)		32 (36)		N/A
Practices represented			12		14		13		N/A
**Usability, mean (SD)^c^**									
	Ease of use and learnability	5.8 (0.8)		5.4 (1.2)		5.9 (0.9)		.50	
	Effectiveness	4.9 (0.8)		4.4 (1.4)		4.5 (1.3)		.30	
	Reliability	2.3 (1.4)		2.5 (1.6)		2.2 (1.3)		.80	
	Satisfaction and future use	5 (1.1)		4.8 (1.7)		4.8 (1.3)		.70	
**Usefulness—child and population health, mean (SD)^d^**									
	Timeliness of care	3.8 (0.7)		3.7 (1)		3.8 (0.9)		.98	
	Equity in access to care	4 (0.8)		3.9 (1)		3.8 (1.1)		.50	
	Family-centeredness of care	3.4 (0.7)		3 (1)		3.1 (1.1)		.30	
	Health of my patients	2.8 (0.7)		3 (0.9)		2.9 (0.9)		.90	
	Continuity of care	3 (0.9)		3.1 (1.2)		3.1 (1)		.80	
	Safety of my patients	2.6 (0.7)		3.1 (1.1)		3 (1)		.20	
**Usefulness—clinician experience, mean (SD)^d^**									
	Financial health of my practice	2.8 (0.9)		2.8 (1)		2.6 (1.1)		.30	
	Sense of accomplishment from my work	2.7 (0.8)		2.6 (1)		2.2 (1)		.07	
	Satisfaction with how I spend my time	2.6 (1.1)		2.5 (1.1)		2.2 (1)		.20	
	Sense of connectedness with patients	2.3 (0.9)		2.6 (1.2)		2.3 (1.2)		.80	
**Suitability of telemedicine for specific reasons, mean (SD)^e^**									
	Acute care	2.2 (0.5)		2.3 (0.5)		2.3 (0.5)		.70	
	Chronic disease management	2.6 (0.7)		2.6 (0.7)		2.5 (0.7)		.70	
	Preventive care	1.9 (0.9)		1.8 (0.8)		1.8 (0.8)		.80	
	Follow-up care	2.4 (0.5)		2.3 (0.7)		2.3 (0.6)		.80	
	Care coordination	3.1 (0.8)		3 (0.7)		2.8 (0.7)		.30	
	Mental health	3.2 (0.7)		3 (0.5)		3 (0.7)		.30	

^a^*P* values reported from clinician survey responses compared across tertiles using linear regression clustered by practice.

^b^N/A: not applicable.

^c^Survey questions answered using a 7-point Likert scale, where 1 indicates “strongly disagree” and 7 indicates “strongly agree.”

^d^Survey questions answered using a 5-point Likert scale, where 1 indicates “much worse,” 3 indicates “about the same,” and 5 indicates “much better.”

^e^Survey questions answered using a 4-point Likert scale, where 1 indicates “never” and 4 indicates “always.”

## Discussion

### Principal Findings

Among a network of pediatric practices, we examined practice-level variation in use of telemedicine during the COVID-19 pandemic, allowing us to explore ongoing questions about the relationship between telemedicine use, receipt of care, and equity in receipt of care. We found that increased practice-level telemedicine use was associated with more physicians in the practice, more varied telemedicine encounter diagnoses, and fewer in-person office visits per 1000 patients per week.

While concerns exist that telemedicine may promote overutilization, we found that in the context of the pandemic, greater pediatric primary care practice-level telemedicine use did not result in overutilization but rather supported primary care encounter volume slightly closer to usual rates during a time when underutilization of primary care and other health care settings was prevalent [24-28]. We also found that high telemedicine use practices had slightly more ED visits than low telemedicine use practices during the study period. Again, this finding must be interpreted in the setting of overall decreased health care encounters during this time, such that this slight increase could represent either a greater ability of telemedicine to triage children to emergent care during the COVID-19 pandemic or, alternatively, a slightly greater need for emergent care for patients in these practices. Another possibility is that high telemedicine use practices may have adhered more strictly to guidelines to reduce in-person office visits; that goal, rather than telemedicine itself, may have led to the slight increase in ED visits.

In addition to concerns about overutilization, concerns have been raised about whether increased telemedicine use will improve equity of access to care or exacerbate disparities in access due to the digital divide [2,29,30]. In our sample, children without a preventive visit in the past year and children who identified as races or ethnicities other than White non-Hispanic were less likely to have had a telemedicine visit, while telemedicine use did not vary by child insurance type. These differences in the overall population use of telemedicine build upon prior concerns about differences in access to broadband internet, patient portals, and telemedicine [29,31-34]. However, the nonsignificant interaction terms indicate that increased practice-level use of telemedicine services during this specific period neither reduced nor increased these differences. In cases where telemedicine is intended to be a tool to improve equity in access to care, these results indicate that simply increasing the use of telemedicine may not be sufficient to ensure more equitable access. Indeed, telemedicine has the potential to exchange one set of barriers to care (transportation issues, time constraints, hidden costs of missing work) for another (need for internet access, device capability, computer literacy). For telemedicine to more effectively reduce disparities in access, it will be necessary to implement telemedicine in ways that more intentionally overcome barriers.

While increased use of telemedicine by practices did not translate into greater equity in telemedicine use for patients, it did translate into more varied telemedicine use based on visit diagnoses. Additionally, we observed an increase in the proportion of visits related to respiratory and ear, nose, and throat (ENT) symptoms. Concerns have been raised about assessments of ears in the absence of tele-otoscopy, which was not available for the studied visits [35]. Given that clinicians in low versus high telemedicine use tertiles reported similar views about the suitability of telemedicine for acute care, increased respiratory and ENT diagnoses at high telemedicine use practices may reflect a stronger practice-wide commitment to reducing in-person visits due to the COVID-19 pandemic as opposed to increased comfort with caring for these diagnoses via telemedicine. For all practices, regardless of their use tertile, the most common telemedicine visit diagnosis categories were mental health and skin-related diagnoses. This differs from the most common diagnoses for visits by children to commercial direct-to-consumer telemedicine, where the most common visit diagnosis category was nose/sinus infections [4]. In contrast, the most common diagnosis group among these primary care telemedicine visits was mental health, which accounted for approximately 30% of telemedicine visits across the full set of practices.

### Limitations

One key limitation of this EHR analysis is we cannot account for patient or parent preferences for or satisfaction with telemedicine use. Additionally, the racial and ethnic diversity within our sample was minimal, limiting analyses by race and ethnicity to a comparison of White non-Hispanic children and children identified as any other race or ethnicity. We also sought to compare visit volume by parental language; however, limited numbers of children with parental preference for non-English languages were identified in the relevant EHR field (0.5%). Although systems are in place to integrate ED and urgent care visit information into the EHR, ED and urgent care visits outside of our health system may still have been missed. However, we do not anticipate that this would result in any systematic bias across the practice tertiles given the large number of integrated ED and urgent care centers across the region. We also note that we focused this analysis on telemedicine visit diagnoses and volumes and did not examine quality of care or clinical outcomes. Finally, this analysis focused on telemedicine use within a specific context; therefore, the generalizability of the findings will need to be examined through other sources.

### Conclusion

This study demonstrates that a large pediatric primary care network rapidly integrated use of telemedicine when given a favorable payment environment and public health mandates. The integration of telemedicine allowed continued contact with patients during the pandemic, largely for mental health care, with high practice-level telemedicine use allowing for more encounters with patients per week during a time where underutilization of primary care was common. Further work is needed to understand the sustainability of the pandemic-related surge in primary care telemedicine use and to identify ways to enhance the ability of telemedicine to improve access for those with access barriers.

## Appendix

Multimedia Appendix 1 Clinician survey.

## Abbreviations

AAP: American Academy of Pediatrics
APP: advanced practice provider
ED: emergency department
EHR: electronic health record
ENT: ear, nose, and throat
HIPAA: Health Insurance Portability and Accountability Act
ICD-10-CM: International Classification of Diseases, Tenth Revision, Clinical Modification
