# Supplementary material for: Practice-Level Variation in Telemedicine Use in a Pediatric Primary Care Network During the COVID-19 Pandemic: Retrospective Analysis and Survey Study
Source: J Med Internet Res. 2020 Dec 18;22(12):e24345. doi: 10.2196/24345 (PMC7752181; doi:10.2196/24345)
Supplement: Multimedia Appendix 1 [file jmir_v22i12e24345_app1.docx]

**Appendix: Clinician Survey**

How many live interactive audio-video telemedicine visits have you completed with patients?

- - 0
  - 1-5
  - 6-25
  - 26-50
  - 51-75
  - >75

Overall, how would you describe your experience using telemedicine in your practice? (Items adapted from Telehealth Usability Questionnaire^21^)

Strongly Disagree (1), Disagree (2), Somewhat Disagree (3), Neutral (4), Somewhat Agree (5), Agree (6), Strongly Agree (7)

1. It was simple to use telemedicine in my practice.
2. It was easy to learn to use telemedicine in my practice.
3. I could become productive quickly using telemedicine in my practice.
4. The way I interact with telemedicine is pleasant.
5. Telemedicine use is simple and easy to understand.
6. This system is able to do everything I would want it to be able to do.
7. I could easily talk to the patient using telemedicine.
8. I could hear the patient clearly using telemedicine.
9. I felt I was able to express myself effectively using telemedicine.
10. Using telemedicine, I could see the patient as well as if we met in person.
11. I think the visits provided over telemedicine are the same as in-person.
12. I feel comfortable communicating with the patient using telemedicine.
13. Telemedicine is an acceptable way to provide health care services.
14. My patients are eager to continue using telemedicine for clinical care.
15. Overall, I am satisfied with my experience using telemedicine.
16. I hope to continue to use telemedicine after the pandemic.

How often do you think telemedicine can deliver high-quality care to patients presenting for each of the following reasons?

Never (1), Sometimes (2), Usually (3), Always (4)

- Acute care (rash, cough)
- Chronic disease management (asthma, migraines)
- Preventative care
- Hospital or ED follow-up care
- Care coordination needs
- Mental/behavioral health

Based on what you know about telemedicine today, if telemedicine were to become a regular part of primary care in the future how do you think it will impact the following:

Much worse (1), Slightly worse (2), About the same (3), Slightly Better (4), Much Better (5)

1. Health of my patients
2. Safety of my patients
3. Timeliness of care for my patients
4. Patient and family-centeredness of care for my patients
5. Equity in my patients’ ability to access care (more access to care for hard to reach patients)
6. Financial health of my practice
7. Satisfaction with how I spend my clinical time
8. Sense of accomplishment from my work
9. Feeling of connectedness with patients
10. Improved continuity of care

What device have you used MOST to conduct telemedicine visits?

- - Office Desktop
  - Office Laptop
  - Personal Desktop
  - Personal Laptop
  - Smartphone
  - Tablet
  - Other (please specify)

Are you a physician or APP?

- - Physician
  - APP

How many years have you been in practice (not including training)?

- - 0-5years
  - 6-10years
  - 10-19years
  - >20years

Please select your primary practice site [List offered]
